# Neuralgic amyotrophy: sensitivity and specificity of magnetic resonance neurography in diagnosis: A retrospective study

**DOI:** 10.1097/MD.0000000000035527

**Published:** 2023-10-27

**Authors:** Luyao Duan, Liyang Zhao, Ying Liu, Yizhe Zhang, Wensong Zheng, Xiaoman Yu, Hongran Liu, Zequn Li, Zhigang Peng, Xiaona Li

**Affiliations:** a Department of Radiology, The Third Hospital of Hebei Medical University, Hebei Province Biomechanical Key Laborary of Orthopedics, Shijiazhuang, Hebei, China.

**Keywords:** brachial neuritis, brachial plexus, electromyography, magnetic resonance neurography, neuralgic amyotrophy

## Abstract

**Background:**

Neuralgic amyotrophy (NA) is a clinically acute or subacute disease. To study the characteristics of brachial plexus magnetic resonance neurography (MRN) in patients with NA, and to explore the clinical application value of MRN combined with electromyography (EMG) in the diagnosis of NA.

**Methods:**

Brachial plexus MRN images of 32 patients with NA were retrospectively analyzed, and their characteristics were investigated. The accuracy, sensitivity and specificity of MRN, EMG, and the combination of the 2 methods for NA diagnosis were compared.

**Results:**

Among the 32 patients with NA, 28 (87.5%) cases of unilateral brachial plexus involvement, 18 (56.3%) cases of multiple nerve roots involvement. In 10 cases, C5 nerve roots were involved alone, and in 9 cases, C5 to C6 nerve roots were involved together. The T2 signal intensity of the affected nerve increased, and 19 cases showed thickened and smooth nerve root edges. Twelve cases showed uneven thickening and segmental stenosis of the involved nerve roots. The diagnostic accuracy, sensitivity, and specificity of MRN for NA were higher than those of EMG. Combining MRN and EMG could improve the sensitivity and specificity of diagnosis.

**Conclusion:**

The main feature of MRN in patients with NA was that it was unilateral brachial plexus asymmetric involvement. The diagnostic effect of MRN was better than that of EMG. The combined diagnosis of MRN and EMG can help clinicians diagnose NA accurately.

## 1. Introduction

Neuralgic amyotrophy (NA) is a clinically acute or subacute disease. The main symptom is severe pain in the upper extremities followed by muscle weakness and atrophy of the upper extremities,^[1]^ which easily missed and misdiagnosed as muscle strain or shoulder arthritis.^[2]^ NA is also known as brachial plexus neuritis or Parsonage-Turner syndrome which can also involve other peripheral nerves, such as the lumbosacral and phrenic nerves, alone or in combination.^[1,3]^ These variant phenotypes lead to the development of neuralgic amyotrophy syndrome.^[4]^ The pathophysiological mechanism of NA is unclear, but it is currently thought to be an interaction between infection, repetitive or strenuous motor function, and individual genetic susceptibility.^[1]^ Intraoperative exploration of NA revealed marked moderate inflammation of the nerves.^[5]^

Electromyography (EMG) plays an important role in the diagnosis of peripheral neuropathy.^[1]^ However, EMG has great limitations and poor diagnostic accuracy, especially in the early stage of the lesion, which may exaggerate the lesion. It could happen to the diagnosis of NA, too. Magnetic resonance neurography (MRN) scan can effectively suppress the background signal, highlight the brachial plexus signal, and has the unique advantage of displaying the entire brachial plexus anatomy. For the early peripheral neuropathy, MRN can identify and show the changes of the lesion at different stages. Although MRN can provide a better choice for clinicians, EMG examination is as usual as the first method to peripheral neuropathy for doctors.^[1]^

This study mainly analyzed the imaging features of NA on MRN and compares the diagnostic efficacy of MRN and EMG to NA, so as to enable more clinicians to accept MRN.

## 2. Methods

### 2.1. Participants

A total of 32 patients with a clinical diagnosis of NA between March 2021 and February 2022 were included in the study. These patients were diagnosed according to the diagnostic guidelines put forward in a 1999 European Neuromuscular Center workshop for hereditary neuromuscular atrophy, regardless of their family history. Inclusion criteria: Acute onset, severe shoulder pain, upper limb muscle weakness after a few days or weeks; Involved unilateral or bilateral brachial plexus nerves, mainly movement disorders, affected limbs may appear muscle atrophy. Exclusion criteria: Trauma history, surgical history, family history; Neuromuscular dysfunction caused by other peripheral neuropathy; There are contraindications for MRI, such as claustrophobia, metal implants, etc. There were 22 males and 10 females; age range 23 to 78 years; and mean age 53.28 ± 15.23 years. MRN and EMG were performed before clinical treatment, and the time of examination was based on the time of onset (3–28 days, 9 ± 7.43 days). For this is a retrospective study, the diagnosis of NA of participants has been made by the neurologist during hospitalization and we reviewed them referring to the inclusion and exclusion criteria. The study was conducted in accordance with the principles of the Declaration of Helsinki, and the study protocol was approved by the local ethics committee. The oral informed consent from all the patients have been obtained but the written informed consents were waived.

### 2.2. MR imaging techniques

MRN was performed using a 3.0T MR system (Ingenia CX, Philips Healthcare, Netherlands). The patient was supine, with the head advanced, and an 8-channel body coil was covered. The MRN sequences were used for all patients, including axial 3D T2-weighted image driven equilibrium infrequence reset pulse (3D-T2WI-DRIVE) for the preganglionic segment of the brachial plexus and coronal 3D-NerveView for the posterior segment of the brachial plexus. The scanning range in 3D-T2WI-DRIVE sequence was from the 4th cervical to the first thoracic (C4–T1), field of view (FOV) = 150 mm × 150 mm, slice gap = −1.5 mm (to obtain the higher interlayer resolution), slice thickness = 3.0 mm. The 3D-NerveView sequence is a heavily T2W turbo spin-echo with a large FOV and small voxel size, archiving higher isotropic resolution. A short TI inversion recovery was used to suppress the fat signal ^[4]^. The scanning range in 3D-NerveView sequence was from the posterior segment of the brachial plexus ganglion to the bundle branch of the middle humerus, FOV = 300 mm × 453 mm, slice gap = −1.2 mm, slice thickness = 2.4 mm. After scanning, maximum intensity projection images were rendered on the workstation to acquire coronal reconstruction images with a thickness of 20 to 25 mm by an imaging technician with more than 3 years of experience.

All imaging studies were diagnosed by a consensus of 2 experienced radiologists. Both participants had more than 16 years of experience in reading MR scans. Both radiologists were blinded to the diagnoses and patient identities. Before the evaluation, the 2 doctors reviewed the literature of NA disease and reviewed the typical cases. Brachial plexus evaluation was performed at a workstation on maximum intensity projection images, by observing from the proximal root region to the terminal branches. Brachial plexus nerve signal and thickness abnormalities (T2-weighted hyperintensity and enlargement) are considered pathological.

### 2.3. Statistical analysis

To study the difference in NA detection rate between MRN and EMG, mean values and standard deviation were calculated for patient age and time from symptom onset to imaging. Frequency data are presented using contingency tables, and pairwise associations between variables were computed using chi-square or Fisher exact tests. *P* < .05 was considered statistically significant. The consistency between clinical diagnosis, MRN and EMG were analyzed using the Cohen statistics (kappa concordance index). The κ values were interpreted according to the guidelines of Landis and Koch: mild (0–0.2), acceptable (0.21–0.4), moderate (0.41–0.6), substantial (0.61–0.8) and almost perfect (0.81–1). Data were analyzed using SPSS v. 26.0 for Windows (IBM, Portsmouth, UK).

## 3. Results

### 3.1. Patient characteristics

Among the 32 patients, 29 patients had unilateral upper extremities, including 16 patients on the left side, 13 patients on the right side, and 3 patients on both upper limbs. The characteristics of all patients are shown in Table 1.

**Table 1:** Patient characteristics.

Symptoms and signs	Numbers
Muscle weakness and atrophy of the upper trunk of the proximal upper limb	18 (56%)
Middle and lower trunk injury of the distal upper limb	9 (28%)
Whole brachial plexus injury of the whole upper limb	5 (16%)
The first symptoms (pain and pain)	18 (56%)
Had predisposing factors (cooling or fatigue)	11 (34%)
No clear predisposing factors	21 (66%)

The first symptoms in 18 (56%) patients were pain and pain severity. Eleven (34%) patients had predisposing factors before onset, of which 4 had a history of cooling, 7 had a history of fatigue, and 21 (66%) had no clear predisposing factors.

### 3.2. MRN features of NA

There were 3 cases of bilateral limb involvement, 28 (87.5%) cases of unilateral brachial plexus involvement, and 1 case of no obvious abnormality. There were 10 cases of double-root involvement. Three or more roots were involved in 4 cases. Among the 3 patients with bilateral involvement, 1 had left C5-C6 with right C6 involvement, 1 right C5-C6 with left C5 involvement, and 1 left C7 with right C6 involvement. All patients showed changes in the postganglionic segment, and no obvious abnormalities were found in the preganglionic segment (Table 2).

**Table 2:** MRN features of NA.

Brachial plexus	Root numbers	Root lesion	Number
One side (28)	Single root (14)	C5	10
		C6	1
		C7	1
		C8	2
	Two roots (10)	C5–C6	5
		C7–C8	3
		C8–T1	2
	Multiple roots (4)	C5–C8	2
		C6–C8	1
		C7–T1	1
Both Sides (3)	Two roots (1)	Left C7/ right C6	1
	Multiple roots (2)	Left C5–C6/right C6	1
		Right C5–C6/ left C5	1

NA = neuralgic amyotrophy.

**Figure 1.**
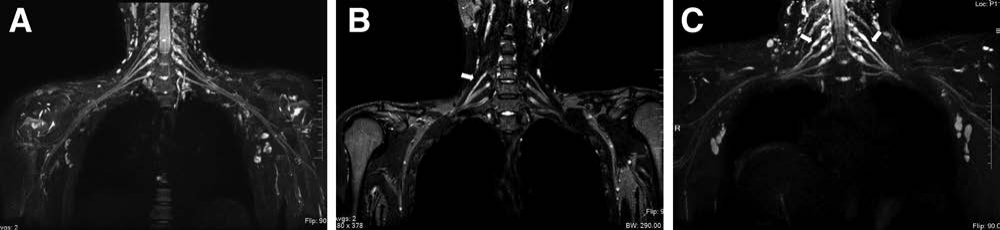
The coronal plane of magnetic resonance neurography in volunteer and patients with neuralgic amyotrophy. (A) Anormal brachial plexus of a 14-year-old female volunteer, nerves were normal in size and sharply define. (B): A male, 31 years old, magnetic resonance neurography showed uniform thickening and increased signal intensity involving the right C5 nerve. (C) A male, 34 years old, magnetic resonance neurography showed segmental thickening and increased signal intensity involving the bilateral C5 nerve roots.

**Figure 2.**
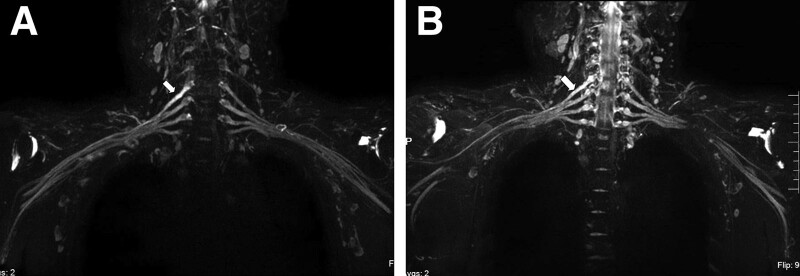
47-year-old male with neuralgic amyotrophy. (A) 40 days after onset, brachial plexus magnetic resonance neurography showed the right C5 (arrow) nerve root was thickened and increased signal intensity. (B) Follow-up 30 days later, comparing with the original image, the signal of the right C5 (arrow) nerve root was lower than before, but still higher than that of surrounding normal nerve.

The coronal reconstruction images of MRN in 31 patients with NA showed that the continuity of the involved nerves was good, and the T2 signal intensity was higher than that of the surrounding normal nerves. Among them, 19 cases showed thickening and smoothing of nerve root edges. Twelve cases showed uneven thickening and segmental stenosis of the involved nerve roots (Fig. 1). A follow-up case in this group showed that after treatment, the signal of the affected nerve was lower than before but still higher than that of the surrounding normal nerve (Fig. 2).

### 3.3. Comparison of the diagnostic value of brachial plexus MRN and EMG

The bilateral brachial plexus nerves were divided into the superior trunk (including C5-6 nerve roots) and the middle and inferior trunk (including C7-T1 nerve roots). Thirty-two patients had 70 sites on the affected side (29 unilateral and 3 bilateral). EMG and MR neurography findings in the patients were not always the same. The specific involvement is shown in Table 3. The κ values for the consistency between clinical diagnosis and MRN was 0.856 (95% confidence interval [CI], 0.735–0.977), indicating almost perfect agreement. The κ values for the consistency between clinical diagnosis and EMG was 0.650 (95% CI, 0.470–0.830), indicating substantial agreement. MR neurography and EMG are shown in Table 4 (*P* = .549 and *P* > .05, respectively). There was no statistically significant difference between MRN and EMG in the diagnosis of NA. The κ values for the consistency between MRN and EMG was 0.683 (95% CI, 0.512–0.854), indicating substantial agreement.

**Table 3:** Involvement of the affected side at 70 sites in 32 neuralgic amyotrophy patients on magnetic resonance neurography and electromyography.

MRN/ EMG	Clinical diagnosis		Total
	Actual Positive	Actual Negative	
Positive	36/34	1/6	37/40
Negative	4/6	29/24	33/30
Total	40/40	30/30	70/70

EMG = electromyography, MRN = magnetic resonance neurography.

**Table 4:** Results of Magnetic resonance neurography and electromyography at 70 sites in 32 neuralgic amyotrophy patients.

MRN	EMG		Total
	Positive	Negative	
Positive	33	4	37
Negative	7	26	33
Total	40	30	70

EMG = electromyography, MRN = magnetic resonance neurography.

### 3.4. Combined diagnosis

A comparison of the accuracy, sensitivity, specificity, misdiagnosis rate, and missed diagnosis rate of MRN combined with EMG for the diagnosis of NA is shown in Table 5. The accuracy, sensitivity and specificity of MRN in diagnosing NA were higher than those of EMG. Compared with MRN, the diagnostic accuracy of Series experiments and Parallel experiments was lower. The diagnostic specificity of Series experiments was 3.43% higher than that of MRN, but the missed diagnosis rate was 7.5% higher. The diagnostic sensitivity of Parallel experiments was 2.5% higher than that of MRN, the rate of misdiagnosis was increased by 20%.

**Table 5:** A comparison of the accuracy, sensitivity, specificity, misdiagnosis rate, and missed diagnosis rate of magnetic resonance neurography combined with electromyography for the diagnosis of neuralgic amyotrophy.

Methods	Accuracy	Sensitivity	Specificity	Missed diagnosis rate	Misdiagnosis rate
MRN	92.86% (65/70)	90.00% (36/40)	96.67% (29/30)	10.00% (4/40)	3.33% (1/30)
EMG	82.86% (58/70)	85.00% (34/40)	80.00% (24/30)	15.00% (6/40)	20.00% (6/30)
Series experiments	90.00% (63/70)	82.50% (33/40)	100.00% (30/30)	17.50% (7/40)	–(0/30)
Parallel experiments	85.71% (60/70)	92.50% (37/40)	76.67% (23/30)	7.50% (3/40)	23.33% (7/30)

EMG = electromyography, MRN = magnetic resonance neurography, NA = neuralgic amyotrophy.

## 4. Discussion

It was reported that the incidence of NA is higher in males, with a male-to-female ratio of approximately 2:1, mainly involving unilateral limbs, and there is no significant difference between the left and right sides.^[6]^ The results of this study showed that among the 32 patients with NA, there were 22 males and 10 were female, with a male-to-female ratio of approximately 2.2:1. Unilateral lesions occurred in 29 (90.63%) patients. Eleven (34.38%) patients had a history of cold and fatigue before onset. Some scholars believe that cold and fatigue are the causes of this disease because they induce autoimmune responses in the body, suggesting that this disease may be related to autoimmune factors.^[1]^

EMG is an important technique for examining the nature, location, and extent of nerve damage.^[7]^The application principle of neuro EMG evoked potential is to record the changes of myoelectric activity during muscle rest and contraction by using nerve evoked potential stimulation, to judge nerve conductance, endplate function, muscle fibers and the innervation of involved nerves, and finally to judge neurogenic injury. The EMG of patients with NA shows mainly neurogenic damage, motor fiber injury is mainly axonal degeneration, motor latency is prolonged, and movement amplitude is significantly decreased. The sensory amplitude is decreased and the sensory conduction velocity is normal.^[8]^

MRN visualizes the structure of nerve bundles by exploiting the differences in water content and connective tissue structure between nerve bundles, nerve membranes, and the peripheral epineurium. To improve the clarity and signal strength of neural displays by suppressing signals from nonneural structures such as fat and blood vessels. The cross-section of the normal axial region is round and oval, the punctate nerve bundles are honeycomb-shaped, and the signal intensity is slightly higher than that of the adjacent muscles. On coronal images, the nerve fibers show slightly low signal intensity, the nerve sheath shows slightly high signal intensity, and the nerve shape shows a “double track” change, gradually tapering from proximal to distal.^[9,10]^

This group of cases was clinically diagnosed with upper trunk injury in 18 cases, with the highest incidence (56.25%). The coronal 3D-NerveView reconstruction image showed that C5 was the most involved. This result is consistent with the findings of Vaishali et al^[11]^ In this group, the involvement of the nerve root mainly showed a higher signal intensity than that of the surrounding normal nerve, which may be due to the infiltration of inflammatory cells and edema of the nerve sheath. In contrast to the surrounding normal nerve, the affected nerves in high signal on MRI were reported to be associated with the disruption of normal axoplasmic flow to increase the proximal and distal axoplasm of the injury site. Some scholars believe that the thickening of the nerve root seen on imaging may be due to an increase in the amount of intraneural fluid caused by venous obstruction and accumulation of fluid in the nerve bundle.^[10]^

With the progression of the disease, nerve self-repair, and intervention of related clinical methods, there will be secondary changes in imaging. Studies have shown that after treatment, with the relief of symptoms, the signal intensity of the injured nerve on MRN decreases but is still higher than that of peripheral normal nerves.^[12,13]^ A follow-up patient in this group also showed similar performance in Figure 2. According to literature reports, clinical follow-up of NA patients found that some patients improved after treatment, but some patients had nerve paralysis, no signs of recovery, and surgical exploration showed thickened edema of the involved nerve, adhesion of peripheral fibers, and hourglass-like stenosis in some segments. These patients need to undergo neurolysis or resection of the stenosis segment to relieve their symptoms.^[14]^ Nagano et al suggested that surgical exploration should be performed in patients with no signs of recovery within 3 months of onset.^[15]^ Brachial plexus MRN can display neuromorphological changes without trauma, which can help clinicians monitor the prognosis of patients with NA and guide them to take appropriate clinical treatment.

The results of this study showed that the accuracy, sensitivity and specificity of MRN for the diagnosis of NA were higher than those of EMG, and the missed diagnosis and misdiagnosis rates were lower than those of EMG. The reasons may be as follows: First, NA has acute or subacute onset, EMG cannot reflect the lesions within 2-3 weeks, and the early diagnosis rate is low.^[16]^ According to the literature, a patient with NA showed thickening of the C7 nerve root on MRN when the course of the disease was 2 weeks, but no obvious abnormality was found on EMG. One month later, EMG showed that the brachial plexus C7 was denervated, which was consistent with the imaging report.^[15]^ Second, NA is characterized by patchy, multifocal involvement, which makes it difficult for EMG to locate specific trunks and branches.^[7]^ MRN provides good visualization of the brachial plexus from the nerve root to branch nerves. When the nerve has inflammatory edema or peripheral fiber adhesions, morphological changes are more intuitive and accurate.^[5]^ In addition, the shape of the brachial plexus is complex, and distal branch nerves are often formed by the intersection of different nerve trunks. In patients with NA, the nerve root is the most common site of involvement and the terminal branch is the least common site of involvement.^[11]^ EMG is more accurate in the diagnosis of distal branch nerves, but it is often difficult to diagnose injury to the high nerve root.

## 5. Limitations

There are still some limitations in this study. Its retrospective nature, the sample size of this study is deemed not adequate. Moreover, when multiple nerve roots are involved at the same time, the degree of the lesion is different, and the signal intensity of MRN may vary greatly, so it is easy to miss the nerve roots whose signal increase is not obvious. A further study of MRN is needed to do in future.

## 6. Conclusion

The main feature of MRN in patients with NA was that it was unilateral brachial plexus asymmetric involvement. The diseased nerve roots were thickened and T2 signals increased. Brachial plexus MRN has high accuracy, sensitivity, and specificity in diagnosing NA, can accurately display the location and morphological changes of brachial plexus injury, can monitor the prognosis of patients, and can guide clinicians to take appropriate treatment. The diagnostic effect of MRN was better than that of EMG. The combined application of MRN and EMG can improve the sensitivity and specificity of diagnosis. This can help clinicians diagnose NA correctly.

## Acknowledgments

We would like to thank the staff of the participants.

## Author contributions

**Conceptualization:** Liyang Zhao, Yizhe Zhang, Wensong Zheng.

**Data curation:** Ying Liu, Xiaoman Yu, Hongran Liu, Zequn Li.

**Formal analysis:** Xiaona Li.

**Funding acquisition:** Zhigang Peng.

**Investigation:** Liyang Zhao, Yizhe Zhang, Wensong Zheng, Xiaoman Yu.

**Methodology:** Ying Liu.

**Project administration:** Zhigang Peng, Xiaona Li.

**Resources:** Xiaoman Yu, Zequn Li.

**Software:** Liyang Zhao, Yizhe Zhang, Wensong Zheng.

**Supervision:** Zequn Li, Zhigang Peng, Xiaona Li.

**Visualization:** Liyang Zhao, Hongran Liu.

**Validation:** Xiaoman Yu, Zhigang Peng, Xiaona Li.

**Writing – original draft:** Luyao Duan.

**Writing – review & editing:** Luyao Duan.
